# Accuracy of a smartphone application for blood pressure estimation in Bangladesh, South Africa, and Tanzania

**DOI:** 10.1038/s41746-023-00804-z

**Published:** 2023-04-17

**Authors:** Charles Festo, Valerie Vannevel, Hasmot Ali, Tigest Tamrat, Getrud J. Mollel, Tsakane Hlongwane, Kaniz A. Fahmida, Kelsey Alland, María Barreix, Hedieh Mehrtash, Ronaldo Silva, Soe Soe Thwin, Garrett Mehl, Alain B. Labrique, Honorati Masanja, Ӧzge Tunçalp

**Affiliations:** 1 Ifakara Health Institute, Dar es Salaam, Dar es Salaam, United Republic of Tanzania; 2 South African Medical Research Council Maternal and Infant Health Care Strategies Unit, Pretoria, South Africa; 3 Research Centre for Maternal, Fetal, Newborn and Child Health Care Strategies, University of Pretoria, Pretoria, South Africa; 4 Department of Obstetrics and Gynaecology, Kalafong Provincial Tertiary Hospital, Pretoria, South Africa; 5 The JiVitA Maternal and Child Health and Nutrition Research Project, Nasirabad, Keranipara, Rangpur, 5400 Bangladesh; 6 UNDP/UNFPA/UNICEF/World Bank Special Program of Research, Development and Research Training in Human Reproduction (HRP), Department of Sexual and Reproductive Health and Research, World Health Organization, Geneva, Switzerland; 7 Center for Human Nutrition, Department of International Health, Johns Hopkins Bloomberg School of Public Health, Baltimore, MD USA; 8 Department of Digital Health and Innovations, World Health Organization, Geneva, Switzerland

**Keywords:** Public health, Medical research, Population screening

## Abstract

Undetected and unmonitored hypertension carries substantial mortality and morbidity, especially during pregnancy. We assessed the accuracy of OptiBP^TM^, a smartphone application for estimating blood pressure (BP), across diverse settings. The study was conducted in community settings: Gaibandha, Bangladesh and Ifakara, Tanzania for general populations, and Kalafong Provincial Tertiary Hospital, South Africa for pregnant populations. Based on guidance from the International Organization for Standardization (ISO) 81,060–2:2018 for non-invasive BP devices and global consensus statement, we compared BP measurements taken by two independent trained nurses on a standard auscultatory cuff to the BP measurements taken by a research version of OptiBP^TM^ called CamBP. For ISO criterion 1, the mean error was 0.5 ± 5.8 mm Hg for the systolic blood pressure (SBP) and 0.1 ± 3.9 mmHg for the diastolic blood pressure (DBP) in South Africa; 0.8 ± 7.0 mmHg for the SBP and −0.4 ± 4.0 mmHg for the DBP in Tanzania; 3.3 ± 7.4 mmHg for the SBP and −0.4 ± 4.3 mmHg for the DBP in Bangladesh. For ISO criterion 2, the average standard deviation of the mean error per subject was 4.9 mmHg for the SBP and 3.4 mmHg for the DBP in South Africa; 6.3 mmHg for the SBP and 3.6 mmHg for the DBP in Tanzania; 6.4 mmHg for the SBP and 3.8 mmHg for the DBP in Bangladesh. OptiBP^TM^ demonstrated accuracy against ISO standards in study populations, including pregnant populations, except in Bangladesh for SBP (criterion 2). Further research is needed to improve performance across different populations and integration within health systems.

## Introduction

Undetected and unmonitored hypertension carries substantial mortality and morbidity risk in the general population^1^. The condition is even more detrimental in the pregnant population where occurrence of hypertension with or without additional complications (hypertensive disorders of pregnancy—HDP) is associated with substantial risk of death and morbidity for pregnant women and their babies^2^. HDP, especially pre-eclampsia and eclampsia, accounts for ~14% of the global burden of maternal mortality and near-misses in developing regions^3^. HDP are particularly devastating, with potentially permanent effects on mothers and infants^4^. With more countries undergoing the obstetric transition, the proportion of indirect causes of maternal mortality and morbidity are also increasing^5^ representing 27.5% of all maternal deaths between 2003–2009, and the second leading cause of maternal morbidity during the antenatal period, as found in a study published in 2016^3,6^. Yet, deaths and severe morbidities from HDP can be prevented through timely detection and prompt management of high blood pressure (BP)^4,7^.

**Fig. 1:**
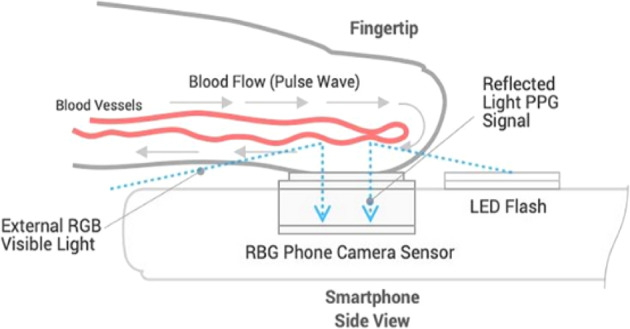
Overview of OptiBP^TM^ (Ref. ^14^).

While BP measurement is recommended as an essential part of all antenatal care (ANC) contacts particularly during the third trimester^8^, the ability to measure with a functioning BP device, recognize elevated numbers, and provide referral when warranted remain important barriers to care. To meet the needs in low- and middle- income country (LMICs) settings, innovations that have the potential to leapfrog and reach populations that do not have access to accurate BP assessment are critical. Digital technologies, particularly propelled by the penetration of mobile phones in LMICs, have been highlighted as a mechanism to address entrenched health system challenges in these settings^9^. OptiBP^TM^, a smartphone application for estimating BP with an emerging research base, was identified in the WHO compendium of innovative health technologies for low-resource settings^10^ as a potential innovation for further exploration and one that did not require external integrations or procurement of equipment beyond a smartphone. The OptiBP^TM^ application runs on Android OS 8.1 and leverages the smartphone camera to record photoplethysmographic (PPG) optical pulse waves derived from blood volume changes at the fingertips (Fig. 1). Users place their finger (oftentimes index finger) on the smartphone camera for 30 s during which they are guided by a timer as the optical pulse waves are extracted. Algorithms transform the optical pulse waves first into raw BP values, which are then processed with a calibration reference to estimate an individual’s BP (see Supplementary Note 1 for more details on the calibration process). In its current state, the OptiBP algorithm requires a one-time baseline BP measurement per individual obtained from a cuff for calibration and subsequently estimating blood pressure.

The algorithms underlying OptiBP^TM^ were first validated in operating room settings using the sensor lens found on pulse oximeters^11,12^. This BP estimation technique was subsequently migrated onto smartphone camera lenses to facilitate more common use. The smartphone application with the OptiBP^TM^ was assessed in Switzerland against both arterial measurements recorded at the fingertips during general anesthesia^13^ and reference auscultatory measurements based on the ISO 81,060–2:2018 standards. These studies demonstrated general concordance of OptiBP with reference measurements and highlighted performance gaps in estimating systolic arterial presure^13–16^. While this research highlighted the potential of OptiBP, these assessments lacked independent evaluations in line with global validation standards and were conducted exclusively in high-income settings.

With the critical demand for innovations to broaden the screening and management of hypertension both for the general and pregnant populations, we conducted a multi-site validation study to assess the accuracy of OptiBP^TM^ across different environmental conditions and demographic profiles. This study was conducted in Tanzania and Bangladesh among general populations, and in South Africa among a pregnant population. The inclusion of pregnant populations was particularly important to align with the study’s key objective of assessing innovations to improve BP screening within routine antenatal care and management of hypertensive disorders of pregnancy. Furthermore, the literature highlighted that cardiovascular and hormonal alterations in pregnancy may affect photoplethysmography signals, which may have implications in detecting pulse waves that inform the smartphone algorithm^17^. The study compared OptiBP^TM^ estimations with reference BP measurements, using a standard auscultatory cuff, in accordance with international regulatory standards for validation of BP devices.

## Results

### Participant characteristics

**Fig. 2:**
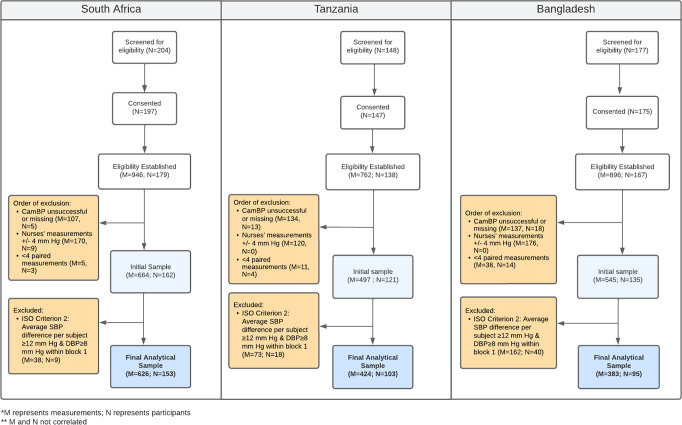
Flow diagram of the client progress (screening, exclusion and analysis) of the CamBP assessment study in three countries (South Africa, Tanzania, and Bangladesh).

The study obtained 626 valid paired measurements across 153 participants in South Africa, 424 valid paired measurements across 103 participants in Tanzania, and 545 valid paired measurements across 95 participants in Bangladesh (Fig. 2). The demographic profile of participants is provided in Tables 1 and 2.

**Table 1:** Characteristics of pregnant population participants—South Africa (*N* = 153; *M* = 626).

Characteristics	*N* (%)
**Age in years**	
18–24	9 (5.8)
25–29	32 (20.7)
30–35	53 (34.2)
36–49	61 (39.4)
**Hemoglobin level (*N*** **=** 90)	
Median (Q1, Q3)	12.4 (11.5,13.5)
**Blood pressure distribution**	
Normotensive (<140/90 mm Hg)	19 (12.4)
Hypertensive without proteinuria (≥140 SBP OR DBP≥ 90 mm Hg)	118 (77.1)
Pre-eclampsia with proteinuria in 24 h and ≥140 SBP OR DBP ≥ 90	16 (10.5)
**Distribution of pregnancy trimesters**	
≤12 weeks	0 (0)
13–28 weeks	76 (49.7)
>28 weeks	77 (50.3)

^a^*SBP* Systolic Blood Pressure, *DBP* Diastolic Blood Pressure.

**Table 2:** Characteristics of general population participants—Tanzania (*N* = 103; *M* = 424) and Bangladesh (*N* = 95; *M* = 383).

Characteristics	Tanzania *N* (%)	Bangladesh *N* (%)
**Age in years**		
18 to 24	7 (6.9)	1 (1.1)
25 to 29	7 (6.9)	1 (1.1)
30 to 35	18 (17.8)	3 (3.2)
36 to 49	37 (36.6)	60 (63.2)
50 to 65	23 (22.8)	22 (23.2)
>65	9 (8.9)	8 (8.4)
Missing	2 (1.9)	0 (0)
**Sex**		
Male	41 (38.6)	37 (38.9)
Female	62 (61.4)	58 (61.1)
Missing	2 (1.9)	0 (0)
**Systolic blood pressure distribution**^a^		
≤100	59 (13.9)	95 (24.8)
101–139	298 (70.2)	219 (57.2)
140–159	55 (13.0)	61 (15.9)
≥160	12 (2.8)	8 (2.1)
**Diastolic blood pressure distribution**^a^		
≤60	29 (6.9)	59 (15.4)
61–85	302 (71.4)	255 (66.6)
85–99	74 (17.3)	63 (16.5)
≥100	19 (4.5)	6 (1.6)

^a^Blood pressure distributions based on measurements (M).

### Performance against ISO criteria

The results for criterion 1 of the analyzed measurements (M) are summarized in Table 3. In South Africa, criterion 1 results show a mean error of 0.5 ± 5.8 mm Hg for the SBP and 0.1 ± 3.9 mmHg for the DBP. In Tanzania, criterion 1 results show a mean error of 0.8 ± 7.0 mmHg for the SBP and −0.4 ± 4.0 mmHg for the DBP. In Bangladesh, criterion 1 results show a mean error of 3.3 ± 7.4 mmHg for the SBP and −0.4 ± 4.3 mmHg for the DBP.

The results for criterion 2 present the average standard deviation of the mean error per subject summarized in Table 4. Across *N* = 153 women in South Africa, criterion 2 shows a SD of 4.9 mmHg for the SBP and 3.4 mmHg for the DBP. Across *N* = 105 individuals in Tanzania, criterion 2 shows a SD of 6.2 mmHg for the SBP and 3.6 mmHg for the DBP. Across *N* = 95 individuals in Bangladesh, criterion 2 shows a SD of 6.4 mmHg for the SBP and 3.8 mmHg for the DBP. There were no adverse events from performing the test or the reference measurement.

**Table 3:** Performance of CamBP device—ISO Criterion 1.

	Pass	Pregnant population South Africa (*M* = 626)	General Population Tanzania (*M* = 424)	General Population Bangladesh (*M* = 383)
**Systolic BP**				
Mean BP differences (mm Hg) for SBP	−5 ≤ mean ≤ 5	0.5 (passed)	0.8 (passed)	3.3 (passed)
SD BP differences (mm Hg)	SD ≤ 8	5.8 (passed)	7.0 (passed)	7.4 (passed)
**Diastolic BP**				
Mean BP differences (mm Hg) for DBP	−5 ≤ mean ≤ 5	0.1 (passed)	−0.4 (passed)	−0.4 (passed)
SD BP differences (mm Hg)	SD ≤ 8	3.9 (passed)	4.0 (passed)	4.3 (passed)

^a^*BP* Blood Pressure, *SD* Standard deviation.

**Table 4:** Performance CamBP device – ISO Criterion 2.

	Pass^a^	Pregnant population South Africa (*N* = 153)	General Population Tanzania (*N* = 103)	General Population Bangladesh (*N* = 95)
**Systolic BP**				
SD of average BP differences	≤6.92^a^ (SA) ≤6.89^a^ (TZ) ≤6.09^a^ (BN)	4.9 (passed)	6.2 (passed)	6.4 (did not pass)
**Diastolic BP**				
SD of average BP differences	≤6.95^a^ (SA) ≤6.93^a^ (TZ) ≤6.93^a^ (BN)	3.4 (passed)	3.6 (passed)	3.8 (passed)

^a^As defined by ISO criterion 2—maximum permissible standard deviation as a function of the error (mmHg). For more details, please refer to table above.

Based on the order of exclusion, 40.7% (466/1144) of excluded measurements were due to nurses’ readings differing by >4 mmHg, followed by 33.0% (378/1144) of exclusions due to unsuccessful CamBP readings. The primary reasons for unsuccessful readings by CamBP were due to the optical wave signals not being captured adequately within the allotted 30 s (63.8% of excluded measurements) or poor-quality signal (19.0% of excluded measurements). Other reasons for exclusion included irregular heart rate (10.3% of excluded measurements), outliers (2.1% of excluded measurements) and missing (4.1% of excluded measurements). In Bangladesh 91.2% (125/137) of the unsuccessful readings also overlapped with cases where ≥4 mmHg differences were observed between nurses’ readings while in South Africa and Tanzania the overlap was <5%.

**Fig. 3:**
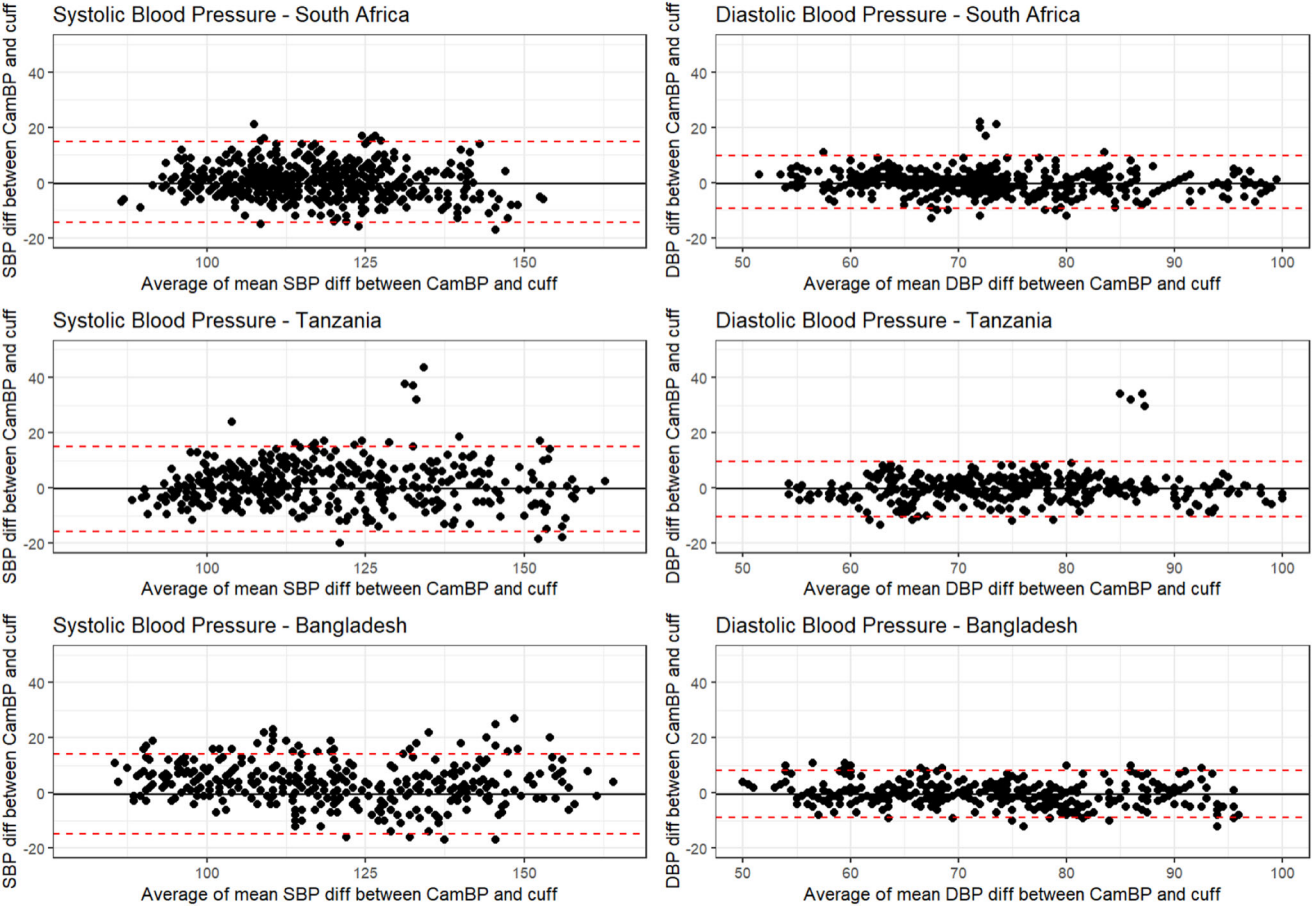
Bland-Altman Plots across countries.

Standardized Bland–Altman scatterplots of the CamBP-reference BP differences against their average values are shown in Fig. 3. The solid line represents the mean error (bias) for each country, whereas the red dotted lines indicate the 95% confidence interval (mean ± 1.96 SD). Across all countries, there are few noticeable outlier differences in BP measurements. Sensitivity analysis comparing actual cuff and screening cuff measurements to CamBP device showing that varied across some subjects, which may be a source of the outliers when comparing cuff and CamBP readings.

## Discussion

This study assessed the accuracy of the OptiBP^TM^ smartphone application (through a blinded version of the application developed for the study called CamBP) to estimate the blood pressure of participants across three LMIC populations (pregnant population in South Africa and, general populations in Tanzania and Bangladesh). All sites achieved ISO 81060–2:2018 criterion 1 for both SBP and DBP. Tanzanian general and South African pregnant populations achieved criterion 2 for both SBP and DBP. Bangladesh’s general population fulfilled criterion 2 for DBP but not SBP. Overall, OptiBP^TM^ demonstrated accuracy in both the general and pregnant populations, except in Bangladesh for SBP (criterion 2). These results expand on the findings from the studies conducted in Switzerland and indicate satisfactory performance in certain populations while highlighting the need for further algorithm refinements to ensure reliability for clinical use.

Global analysis of the burden of hypertension reported a decline in prevalence and increase in treatment rates among high-income countries but an increase in the burden of hypertension in LMICs, where there might also be underestimation due to poorer health systems for detection and initiation of treatment^18^. Low-cost and accessible interventions with minimal maintenance and observer error are of paramount importance to improve timely detection of hypertension among the general population and pregnant women in such settings. In this light, the use of increasingly ubiquitous mobile devices provides novel approach to address un-met needs of early detection and monitoring of hypertension.

While smartphone-based tools present opportunities for addressing health system challenges, the diversity of the participants is a critical factor for studies evaluating technologies that rely on algorithms, such as OptiBP^TM^. Recent developments in machine learning and algorithm development demonstrate that lack of diversity in the training data can lead to biases among groups, and it is encouraged to include affected populations during the initial design^19^. This is particularly important as such types of technologies, known as software as a medical device, or the use of software used for medical purposes without being part of a hardware medical device^20^, use data and algorithms to provide clinical inputs. The conduct of this study in Asia and Africa ensures the underlying algorithms are robust and inclusive of these different populations, as a supplement to the research being done in Europe and North America^14,16^. Furthermore, recent research highlighting variability in the accuracy of devices employing pulse oximetry technology, with detrimental results for non-White populations, depict the importance of accounting for diverse populations in testing and use of medical devices^21^. To our knowledge this is one of the first studies on algorithm-based blood pressure estimation applications conducted in both urban and rural contexts of low- and middle-income settings, where data on medical device validation studies is limited.

The lack of independent evaluations and insufficient rigor in the design of accuracy assessments is also a commonly cited criticism, with ramifications for regulatory oversight and trust by end-users^22–25^. Therefore, this study was developed in accordance with ISO requirements, which entailed a rigorous threshold for inter-nurse agreement to establish the reference value and ensuring a specific distribution of participants across sex, age, and BP parameters. The blinding of all stakeholders, including the study team and software developers, to the device BP values, lends to the credibility of the findings. Furthermore, the data analysis being conducted independently of the software development team seeks to overcome this common pitfall of accuracy assessments.

The continuous evolution of ISO guidance for validation of BP devices and gaps in existing guidance specific to software as a medical device is currently a challenge for accuracy assessments. To account for this, we assembled an independent data review committee, with panelists that were part of the ISO committee or had participated in BP validation studies, to review findings from the learning phase and provide considerations to enhance the study. Furthermore, the planning phase informed the standardization of study procedures and refinement of the OptiBP^TM^ algorithm, which is a step that could be applied to similar studies in the future. Our efforts also highlighted the need for developing appropriate evaluation methodologies and standards that can adapt to the evolving pace of such innovations.

The study also encountered several challenges at the implementation level, which are worth mentioning to inform future efforts. One of which included recruiting participants within the high BP (hypertensive, diastolic ≥100 mmHg, and systolic ≥ 160 mmHg) range required by ISO. Known hypertensives in the community were medicated, which made their BPs either lower than cut-off or controlled. As this challenge was detected during the learning phase, each site introduced mitigation procedures for targeted recruitment of hypertensive populations, which was cleared by ethical review committees prior to the accuracy assessment. Despite this, Bangladesh did not achieve the 5% hypertensive participants needed, which could be a limitation of generalizing the results to the hypertensive population in rural community of Bangladesh. Additionally, the Bangladesh site identified during the routine study data monitoring that measurements for some participants recruited during colder weather were excluded as “OptiBP unsuccessful” (Fig. 2). After noting this, the research team overcame this challenge by instructing participants to warm the finger by gently rubbing both hands to warm fingers and increase the blood flow. Learning from this finding could be considered for further understanding of the limitations of the software.

Lastly, across all three countries, the COVID-19 pandemic affected the implementation of this study and delayed the start of data collection. Study teams took on the necessary preventive measurements such as screening of COVID-19 symptoms, frequent hand washing, wearing masks and social distancing, as well as the WHO team conducting the training on study procedures virtually.

This multisite study contributes to the nascent body of literature on software as a medical device and represents one of the several studies to generate evidence using ISO guidance. Considering the novelty of this technology, future research is still needed to expand understanding on the performance of the application, as well as the health system implications of introducing this innovation across different cadres of health workers in facility and community settings, and to individuals directly as part of a self-care intervention approach. In their current form, the algorithms require an initial/one-time calibration procedure of entering a baseline value from a cuff as part of the process to estimate BP values for each user during the first time OptiBP^TM^ is used, which is a limitation to ensuring standalone use in LMIC settings. Further studies are needed to understand the duration of the initial calibration value and potential of OptiBP^TM^ operating without any initial calibration. This is currently being explored separately by the OptiBP^TM^ development team to minimize the need for recurrent calibration and assess the reliability of using the application as a screening tool without inputting a baseline calibration value. In addition, the conditions affecting OptiBP^TM^ signal quality and successful readings need to be further examined with a wider set of variables. To date, populations with vascular malformation, Raynaud syndrome, and users with damaged/injured fingertip skin are not able to make use of OptiBP due to the technology’s reliance of adequate optical pulse wave signals at the fingertip. Detailed analyses on different subgroup characteristics, such as pregnant women with anemia, age, sex may also provide greater insights on the performance, and further research is needed to explore population, morphological, and external factors affecting fidelity of use For example, environmental factors, such as what was identified during the cold weather in Bangladesh, and technical factors, such as the types of smartphone devices that OptiBP^TM^ could operate on, will require further exploration to ensure accessibility across different global markets. Furthermore, additional work is required to ensure sustainable business models and appropriate data and privacy provisions for such software-based solutions to be deployed and maintained at country level.

With the global penetration of mobile devices and the emergence of digital tools that now offer the capabilities of medical devices, ensuring the clinical validity and safety in accordance with regulatory standards is paramount^19,22,24,26^. Ultimately, innovations, such as OptiBP^TM^ will also require a health system and societal perspective to facilitate their equitable and trusted use, maximize impact, and realize their potential in accelerating progress towards the sustainable development goals.

## Methods

### Study design

This was an observational validation study in which we compared BP measurements taken by two independent trained nurses on a standard auscultatory cuff compared to the BP measurements taken by a smartphone application (a study participant placing his/her right index finger on the camera of a smartphone). For the purposes of the study, we used a research version of OptiBP^TM^ called CamBP, which incorporates the same algorithms and functionality of OptiBP^TM^ but blinds research nurses and research assistants to the BP values generated by the application. The assessment methods and study procedures were based on the guidance from the Consensus Statement published by the Association for the Advancement of Medical Instrumentation, the European Society of Hypertension, the International Organization for Standardization (AAMI/ESH/ISO)^27,28^ and ISO 81,060–2:2018 standards for validation of BP measurement devices^27,28^.

As this was the first time OptiBP^TM^ was assessed in LMIC settings, we included a planning phase to prepare for the study. This included testing the electronic Case Report Form (e-CRF) and device configurations for CamBP, refining the manual of operations to standardize training and procedures across all sites, and applying collected data to train the CamBP algorithms. The planning phase resulted in an update to the CamBP algorithms, used as the final version for the assessment. In addition, an independent data review panel comprised of experts in BP validation, digital health, and obstetric care reviewed findings from the planning phase and provided recommendations that informed the validation study procedures.

The study was conducted in community settings of Gaibanda, Bangladesh and Ifakara, Tanzania for the general population, and Kalafong Provincial Tertiary Hospital outside Pretoria, South Africa for pregnant populations. Johns Hopkins University-JiVita Bangladesh led the Bangladesh site; Ifakara Health Institute led the Tanzanian site, and University of Pretoria Research Centre for Maternal, Fetal, Newborn and Child Health Care Strategies / South African Medical Research Council led the South African site. Data collection began in November 2021 across all three sites and was completed in mid-December 2021. Study teams conducted weekly monitoring of BP distributions to target the recruitment and achieve the ISO sampling requirements.

### Participants

Eligibility was based on age 18–80 years and providing written informed consent; in addition, current pregnancy was a requirement for the enrollment in South Africa (age 18–50 years). Individuals were excluded if they had a BP difference between two arms >15 mmHg for systolic and >10 mmHg for diastolic as measured by cuff; an unstable cardiac condition or in need of oxygen therapy; were unable to place index or middle finger of right hand on smartphone camera; or if their resting BP was >160/110 for pregnant populations and 180/120 for general population. Individuals excluded for extreme BP were also referred for immediate care or managed within the hospital setting in South Africa. Bangladesh also conducted additional screening to exclude individuals with COVID-19 symptoms. Pregnant individuals were excluded from the general population recruitment in Tanzania and Bangladesh.

In Bangladesh, participants were recruited from a pool of community health workers (CHWs) and their spouses in the catchment area. To limit COVID-19 exposure, the study team contacted the CHWs to obtain preliminary consent over the phone, screened for COVID-19 symptoms and made an appointment for participants to come to the study center for potential enrollment and written consent. Towards the end of the study, the team employed snowball sampling methods to identify individuals for meeting the required BP distributions.

In Tanzania, participants were recruited from five wards in Ifakara town by asking individuals to come to a designated place (e.g., school, village government office) at a set date and time. Messages were communicated through radio, town meetings and local leaders. One week prior to the beginning of data collection, the Ifakara Health Institute team conducted a meeting with ward executive officers and co-developed an implementation plan together with the local leaders to determine identification of venues for data collection. Local leaders led the coordination and invitation of community members to selected venues for data collection on planned dates. Nurses screened individuals for exclusion criteria and informed consent. Additionally, to obtain the BP distributions, study teams identified participants from the Heart and Lung clinic in Ifakara for eligibility screening and possible enrollment into the study.

In South Africa, pregnant women across all trimesters attending ANC clinic at Kalafong hospital were recruited after being assessed by a physician for their routine ANC. Data collectors approached women until the target total of consenting and eligible women for that day was reached. Once the required sample for normotensive pregnant women was reached, the study recruited only women with hypertension. Pregnant women with hypertension were then assessed for pre-eclampsia. Recruitment of women with hypertension continued until the target sample of pre-eclamptic women was reached. To be more efficient in identifying potential pre-eclamptic participants, the study team also recruited from the antenatal ward.

Preliminary screening was conducted to ensure the individual had not participated in the planning phase, did not have an absence of fingers or limbs, or major heart conditions. Eligibility was established after informed consent and a more detailed screening of blood pressure, pulse, and oxygen saturation.

### Sample size

Sample size calculations were based on recommendations from the Collaboration Statement published by the Association for the Advancement of Medical Instrumentation, the European Society of Hypertension, the International Organization for Standardization (AAMI/ESH/ISO)^27,28^. This statement suggests a sample size of at least 85 participants for general population, as well as sample size of 45 participants for pregnant women, if an independent general population 85-subject study has been completed successfully^27,28^. In accordance with these recommendations, the sample size was set to 100 general participants each for the analytical sample in both Tanzania and Bangladesh, and 60 pregnant participants for the analytical sample in South Africa. In addition, the analytical sample for the general population participants needed to demonstrate a minimum 30% participation rate of both sexes and the following BP distributions: at least 5% systolic ≤100 mmHg, 5% systolic ≥160 mmHg; at least 20% systolic ≥140 mmHg; at least 5% diastolic ≤60 mmHg; at least 5% diastolic ≥100 mmHg; and at least 20% diastolic ≥85 mmHg^27,28^. For pregnant populations, the analytical sample needed to be distributed along the following groups with a minimum of 20 participants in each group: (i) normotensive pregnant women <140/90 mmHg; (ii) hypertensive pregnant women without proteinuria >300 mg in 24 h and BP ≥140/90 mmHg; and (iii) pre-eclampsia, with proteinuria >300 mg in 24 h and DBP ≥90 mmHg^27,28^.

### Procedures

Each site’s data collection team comprised of one research assistant and two nurses. The teams received a 3-day training on the study procedures, which included components on the protocol, standardizing BP measurement, and study workflow. This training was conducted twice, prior to the planning phase and refresher training prior to this study. As part of the quality assurance and training to standardize BP measurement prior to data collection, each pair of nurse teams had to achieve inter-rater reliability of systolic BP (SBP) and diastolic BP(DBP) differences ≤5 mmHg for 45 out of 50 practice measurements and SBP and DBP differences between ≤10 mmHg for 48 out of 50 practice measurements.

The study used two data collection tools: an e-CRF installed on tablets and the CamBP application installed on Samsung S7 smartphones. The e-CRF was developed using the Open Data Kit (ODK) mobile data collection platform. The e-CRF collected eligibility requirements, informed consent, sex, demographic information, finger condition, medical history, and reference (cuff) BP measurements. The CamBP application collected information on the sex of the participant and an initial calibration value derived from the cuff. The CamBP application used this information to estimate BP values and generate the index test measurements. These BP measurements were recorded on the smartphone and transmitted to a server to produce a spreadsheet of the recorded measurements. A unique identifier was preassigned to all participants and used to link the data from the two data collection tools. The study team, software developers and participants were all blinded to the CamBP outputs at the time of data collection. The software developers also did not have access to the reference blood pressure readings to ensure independent analysis.

During data collection, reference measurements were based on the average of two nurses’ simultaneous BP measurements taken through a manual double stethoscope (BV Medical Teaching/Training Dual-Head Stethoscope). Nurses were blinded to each other’s measurement readings. A research assistant was tasked with recording the reference measurements into the e-CRF. Based on ISO 81060–2 criteria, each round of nurses’ values were considered as valid paired readings and averaged to derive the reference value for the round if their measurements did not differ by >4 mmHg^27,28^. The study required a minimum of four rounds with valid paired readings with inter-nurse agreement and successful signal capture from the application. In instances where the inter-nurse difference was >4 mmHg, the e-CRF calculations discarded measurements and the research assistant notified nurses to repeat rounds. Rounds could be repeated up to four times, with a total maximum of eight rounds per participant (Table 5)^27,28^. The readings taken of the participants represented the resting state (after at least 5 min of sitting) to fulfill the ISO requirements.

**Table 5:** Workflow for study participation.

**Participant sits in a chair and relaxes for 5** **min**	
**Calibration round**	• Take BP measurement with manual cuff by 2 nurses
	• Take CamBP measurement
	• Conduct up to 6 times for 2 valid CamBP readings
**Validation BP measurements for accuracy evaluation**	
Round 1	• Take 1st reference BP measurement with manual cuff by 2 nurses (R1)
	• Take 1st test CamBP measurement (T1)
Round 2	• Take 2nd reference BP measurement with manual cuff by 2 nurses (R2)
	• Take 2nd test CamBP measurement (T2)
Round 3	• Take 3rd reference BP measurement with manual cuff by 2 nurses (R3)
	• Take 3rd test CamBP measurement (T3)
Round 4	• Take 4th reference BP measurement with manual cuff by 2 nurses (R4)
	• Take 4th test CamBP measurement (T4)
Round 5–8	• Repeat above as necessary to account for rounds with inter-nurse difference ≥4 mmHg, for up to four additional rounds

### Data management

From the full dataset with eligible participants and BP measurements, several exclusion criteria were applied based on ISO 81060–2 guidance (Fig. 2). Firstly, exclusions due to CamBP device failure (e.g., not capturing optical pulse waves within allotted time, poor quality of the signal, irregular heart rate, outlier) were dropped. Secondly, measurements with inter-nurse differences in BP readings of >4 mmHg were excluded. Thirdly, participants with less than three paired measurements were also excluded. Lastly, where the participant’s systolic BP differed by >12 mmHg or >8 mmHg in diastolic BP across 2 out of the 4 rounds of data collection, the study participant and all their respective measurements were excluded as per the Collaboration Statement^27^.

### Statistical analysis

Univariable analysis was conducted to describe age groups, BP measurements, and sex. Analysis was conducted for each country separately. Age groups were categorized based on both a general (18–24, 25–29, 30–35, 36–49, 50–65, >65 years) and pregnant population distributions (18–24, 25– 29, 30–35, 36–49 years). For the pregnant population, the median and interquartile ranges of the hemoglobin values are described.

The analysis focused on measurements assessed against ISO 81060–2 criterion 1 and 2^27,28^. For criterion 1, the mean error and standard deviation between the CamBP device value and the corresponding reference cuff value was estimated for each valid round of BP measurements. Results of the analysis were compared against the ISO 81060–2:2018 criterion 1 standard, in which the requirement for the device measurement to be validated by the cuff measurement is to observe a mean error of ≤±5 mmHg with standard deviation of the mean error of ≤ 8 mmHg^27,28^.

For criterion 2, each subject’s BP measurements were averaged. Subsequently, the standard deviation of the difference between the subject’s average BP measurement from CamBP and corresponding cuff values was derived. These values were evaluated against the maximum permissible standard deviation as function of the estimated mean error (mmHg) calculated from criterion 1 (Table 6)^27,28^.

**Table 6:** ISO Criterion 2—Maximum permissible standard deviation as function of the error (mmHg).

Mean error	0,0	0,1	0,2	0,3	0,4	0,5	0,6	0,7	0,8	0,9
**±0**,	6.95	6.95	6.95	6.95	6.93	6.92	6.91	6.90	6.89	6.88
**±1**,	6.87	6.86	6.84	6.82	6.80	6.78	6.76	6.73	6.71	6.68
**±2**,	6.65	6.62	6.58	6.55	6.51	6.47	6.43	6.39	6.34	6.30
**±3**,	6.25	6.20	6.14	6.09	6.03	5.97	5.89	5.83	5.77	5.70
**±4**,	5.64	5.56	5.49	5.41	5.33	5.25	5.16	5.08	5.01	4.90
**±5**,	4.79	–	–	–	–	–	–	–	–	–

Example of how to read ISO criterion 2 table: for mean error of ±4,2 mmHg the maximum permissible standard deviation is 5,49 mmHg.

The protocol was approved by the WHO/HRP Research Review Panel. Ethical approval for both the planning phase and accuracy assessment and was obtained from the WHO Ethical Review Committee (Protocol A65932) as well as the following relevant national entities (Bangladesh Medical Research Council—(BMRC/NREC/2016–2019/07); University of Pretoria Faculty of Health Sciences ethics Research Ethics Committee (626/2018)—South Africa, NIMR/R.81/Vol.IX/3159—Tanzania).

All analyses were conducted in SAS version 9.4 (Copyright © 2016 by SAS Institute Inc., Cary, NC, USA) and R version 4.1.1 (R Core Team 2021. R Foundation for Statistical Computing, Vienna, Austria. https://www.R-project.org/).

### Reporting summary

Further information on research design is available in the Nature Research Reporting Summary linked to this article.

## Supplementary information

Supplementary Note 1.

REPORTING SUMMARY

## Data Availability

All study materials will be available upon request. Anonymized data will be made available towards regulatory approval after publication of findings with permission from country teams. Access to de-identified dataset and study materials, including the protocol, statistical analysis plan, and case reporting forms, may be made available based on email request to SRHHRP@who.int, using a data agreement; please indicate “CamBP research study” in the subject line.

## Code availability

The statistical code for analysis may be made available based on email request to SRHHRP@who.int, using a code availability agreement; please indicate “CamBP research study” in the subject line.
