# Supplementary material for: Accuracy of a smartphone application for blood pressure estimation in Bangladesh, South Africa, and Tanzania
Source: NPJ Digit Med. 2023 Apr 17;6:69. doi: 10.1038/s41746-023-00804-z (PMC10107587; doi:10.1038/s41746-023-00804-z)
Supplement: Supplementary file 1 — Supplementary Note 1. [file 41746_2023_804_MOESM1_ESM.pdf]

Supplementary Note 1. Background on the algorithm processing (extracted from OptiBP technical documentation in investigator brochure, 2021)

OptiBP is embedded into a mobile application that can be installed on smartphones. The mobile application interacts with the smartphone to access the device camera and capture the date. The core of the mobile application is the algorithm that estimates BP with the following steps:

- Camera captures optic recordings that are extracted as “raw values”
- Raw value is compared with reference/baseline BP value and establish a calibration reference
- Raw value is processed with the calibration reference to estimate BP

The application provides the following interfaces:

- Inputs:
    - Subject login (ID, age, gender, height, weight)
    - Start/Stop blood pressure estimation
    - Reference measurements (one-time calibration)
- Outputs:
    - Estimated diastolic blood pressure
    - Estimated systolic blood pressure

Fingertip PPG / optical pulse waves capture the synchronous cardiac variations in blood volume at the fingertip that rise from heartbeats. The blood volume change happens in the arterioles of the fingertip, and the signal acquisition captures the pulsative (AC) component of the PPG signal. The signal acquisition has been designed to remove the incidence of the different artifacts (motion, ambient light) and the various superimposed (DC) components shaped by respiration, sympathetic nervous system activity, and thermoregulation. As the technology relies on optical pulse wave signal at the fingertip, the Furthermore, as the technology relies on optical pulse wave signal at the fingertip, a documented limitation is that populations with vascular malformation, Raynaud syndrome, and users with damaged/injured fingertip skin are not able to make use of OptiBP.
