# Supplementary material for: Accuracy of a smartphone application for blood pressure estimation in Bangladesh, South Africa, and Tanzania
Source: NPJ Digit Med. 2023 Apr 17;6:69. doi: 10.1038/s41746-023-00804-z (PMC10107587; doi:10.1038/s41746-023-00804-z)
Supplement: Supplementary file 2 — REPORTING SUMMARY [file 41746_2023_804_MOESM2_ESM.pdf]

nature portfolio

Corresponding author(s): Tamrat
Last updated by author(s): Jan 10, 2023

Reporting Summary

Nature Portfolio wishes to improve the reproducibility of the work that we publish. This form provides structure for consistency and transparency in reporting. For further information on Nature Portfolio policies, see our Editorial Policies and the Editorial Policy Checklist.

Statistics

For all statistical analyses, confirm that the following items are present in the figure legend, table legend, main text, or Methods section.

n/a	Confirmed	
☐	☒	The exact sample size (*n*) for each experimental group/condition, given as a discrete number and unit of measurement
☐	☒	A statement on whether measurements were taken from distinct samples or whether the same sample was measured repeatedly
☐	☒	The statistical test(s) used AND whether they are one- or two-sided<br>*Only common tests should be described solely by name; describe more complex techniques in the Methods section.*
☐	☒	A description of all covariates tested
☒	☐	A description of any assumptions or corrections, such as tests of normality and adjustment for multiple comparisons
☐	☒	A full description of the statistical parameters including central tendency (e.g. means) or other basic estimates (e.g. regression coefficient) AND variation (e.g. standard deviation) or associated estimates of uncertainty (e.g. confidence intervals)
☒	☐	For null hypothesis testing, the test statistic (e.g. *F*, *t*, *r*) with confidence intervals, effect sizes, degrees of freedom and *P* value noted<br>*Give P values as exact values whenever suitable.*
☒	☐	For Bayesian analysis, information on the choice of priors and Markov chain Monte Carlo settings
☒	☐	For hierarchical and complex designs, identification of the appropriate level for tests and full reporting of outcomes
☒	☐	Estimates of effect sizes (e.g. Cohen's *d*, Pearson's *r*), indicating how they were calculated

Our web collection on statistics for biologists contains articles on many of the points above.

Software and code

Policy information about availability of computer code

Data collection	The study used two data collection tools: an e-CRF installed on tablets and the CamBP application installed on Samsung S7 smartphones. The e-CRF was developed using the Open Data Kit (ODK) mobile data collection platform. The e-CRF collected eligibility requirements, informed consent, demographic information, finger condition, medical history and reference (cuff) BP measurements. The CamBP application collected information on the sex of the participant and an initial calibration value derived from the cuff. The CamBP application used this information to estimate BP values and generate the index test measurements. These BP measurements were recorded on the smartphone and transmitted to a server to produce a spreadsheet of the recorded measurements. A unique identifier was preassigned to all participants and used to link the data from the two data collection tools. The study team, software developers and participants were all blinded to the CamBP outputs at the time of data collection. The software developers also did not have access to the reference blood pressure readings to ensure independent analysis.
Data analysis	All analyses were conducted in SAS version 9.4 (Copyright © 2016 by SAS Institute Inc., Cary, NC, USA) and R version 4.1.1 (R Core Team 2021. R Foundation for Statistical Computing, Vienna, Austria. https://www.R-project.org/).

For manuscripts utilizing custom algorithms or software that are central to the research but not yet described in published literature, software must be made available to editors and reviewers. We strongly encourage code deposition in a community repository (e.g. GitHub). See the Nature Portfolio guidelines for submitting code & software for further information.

Data

Policy information about availability of data

All manuscripts must include a data availability statement. This statement should provide the following information, where applicable:

- Accession codes, unique identifiers, or web links for publicly available datasets
- A description of any restrictions on data availability
- For clinical datasets or third party data, please ensure that the statement adheres to our policy

All study materials will be available upon request. Anonymized data will be made available towards regulatory approval after publication of findings with permission from country teams. Access to de-identified dataset and study materials, including the protocol, statistical analysis plan, and case reporting forms, may be made available based on email request to SRHHRP@who.int, using a data agreement; please indicate “CamBP research study” in the subject line.

Human research participants

Policy information about studies involving human research participants and Sex and Gender in Research.

Reporting on sex and gender	The study followed the ISO requirements for a minimum 30% participation rate of both male and female sexes. The e-CRF collected eligibility requirements, informed consent, sex, demographic information, finger condition, medical history and reference (cuff) BP measurements. The CamBP application collected information on the sex of the participant and an initial calibration value derived from the cuff. Sex was collected for the general population arms (Bangladesh and Tanzania), but not collected for the South Africa arm as it was only conducted on pregnant populations. Sex is reported as part of the participant characteristics. Gender was not collected.
Population characteristics	For the general population, the following population characteristics were collected:<br>-sex<br>-age/date of birth<br>-height<br>-weight<br>-cardiac related health conditions<br>-blood pressure and cardiac related medications<br>-use of tobacco products and other substance use<br>-finger size<br>-presence of callouses or scars on finger used for CamBP<br><br>With the exception of sex, the same questions were asked for the pregnant population in South Africa. In addition, the following characteristics were collected:<br>-gestational age in weeks and days<br>-hypertension history<br>-hemoglobin<br>-presence of protein in urine for assessing for pre-eclampsia
Recruitment	In Bangladesh, participants were recruited from a pool of community health workers (CHWs) and their spouses in the catchment area. In Tanzania, participants were recruited from five wards in Ifakara town by asking individuals to come to a designated place (e.g., school, village government office) at a set date and time. Messages were communicated through radio, town meetings and local leaders. In South Africa, pregnant women attending ANC clinic at Kalafong hospital were recruited after being assessed by a physician for their routine ANC. Data collectors approached women until the target total of consenting and eligible women for that day was reached. Once the required sample for normotensive pregnant women was reached, the study recruited only women with hypertension.
Ethics oversight	The protocol was approved by the WHO/HRP Research Review Panel. Ethical approval for both the planning phase and accuracy assessment and was obtained from the WHO Ethical Review Committee (Protocol A65932) as well as the following relevant national entities (Bangladesh Medical Research Council- (BMRC/NREC/2016-2019/07); University of Pretoria Faculty of Health Sciences ethics Research Ethics Committee (626/2018)) – South Africa, NIMR/R.81/Vol.IX/3159 -Tanzania).

Note that full information on the approval of the study protocol must also be provided in the manuscript.

Field-specific reporting

Please select the one below that is the best fit for your research. If you are not sure, read the appropriate sections before making your selection.

☒ Life sciences ☐ Behavioural & social sciences ☐ Ecological, evolutionary & environmental sciences

For a reference copy of the document with all sections, see nature.com/documents/nr-reporting-summary-flat.pdf

Life sciences study design

All studies must disclose on these points even when the disclosure is negative.

Sample size	Sample size calculations were based on recommendations from the Collaboration Statement published by the Association for the Advancement of Medical Instrumentation, the European Society of Hypertension, the International Organization for Standardization (AAMI/ESH/ISO).18,19 This statement suggests a sample size of at least 85 participants for general population, as well as sample size of 45 participants for pregnant women, if an independent general population 85-subject study has been completed successfully. In accordance with these recommendations, the sample size was set to 100 general participants each for the analytical sample in both Tanzania and Bangladesh, and 60 pregnant participants for the analytical sample in South Africa. In addition, the analytical sample for the general population participants needed to demonstrate a minimum 30% participation rate of both sexes and the following BP distributions: at least 5% systolic ≤ 100 mmHg, 5% systolic ≥ 160 mmHg; at least 20% systolic ≥ 140 mmHg; at least 5% diastolic ≤ 60 mmHg; at least 5% diastolic ≥ 100 mmHg; and at least 20% diastolic ≥ 85 mmHg.18,19 For pregnant populations, the analytical sample needed to be distributed along the following groups: (i) normotensive pregnant women < 140/90 mmHg; (ii) hypertensive pregnant women without proteinuria > 300 mg in 24 h and BP ≥ 140/90 mmHg; and (iii) pre-eclampsia, with proteinuria > 300 mg in 24 h and DBP ≥ 90 mmHg.
Data exclusions	From the full dataset with eligible participants and BP measurements, several exclusion criteria were applied based on ISO 81060-2 guidance .Exclusions due to CamBP device failure (e.g., not capturing optical pulse waves within allotted time, poor quality of the signal, irregular heart rate, outlier) were dropped. Secondly, measurements with inter-nurse differences in BP readings of greater than 4 mmHg were excluded. Thirdly, participants with less than three paired measurements were also excluded. Lastly, where the participant's systolic BP differed by more than 12 mmHg or more than 8 mmHg in diastolic BP across 2 out of the 4 rounds of data collection, the study participant and all their respective measurements were excluded as per the Collaboration Statement.
Replication	The study included a planning phase to prepare for the study. This included testing the electronic Case Report Form (e-CRF) and device configurations for CamBP, refining the manual of operations to standardize training and procedures across all sites, and applying collected data to train the CamBP algorithms. As part of the quality assurance and training to standardize BP measurement prior to data collection, each pair of nurse teams had to achieve inter-rater reliability of systolic BP (SBP) and diastolic BP(DBP) differences ≤5mmHg for 45 out of 50 practice measurements and SBP and DBP differences between ≤10mmHg for 48 out of 50 practice measurements. During data collection, reference measurements were based on the average of two nurses' simultaneous BP measurements taken through a manual double stethoscope. Nurses were blinded to each other's measurement readings. A research assistant was tasked with recording reference measurements into the e-CRF. Based on ISO 81060-2 criteria, nurses' values that did not differ by more than 4mmHg were considered as valid paired readings for averaging.
Randomization	Allocations were not random as there was a need to meet different ISO blood pressure distributions to demonstrate at least 5% systolic ≤ 100 mmHg, 5% systolic ≥ 160 mmHg; at least 20% systolic ≥ 140 mmHg; at least 5% diastolic ≤ 60 mmHg; at least 5% diastolic ≥ 100 mmHg; and at least 20% diastolic ≥ 85 mmHg.18,19 For pregnant populations, the analytical sample needed to be distributed along the following groups: (i) normotensive pregnant women < 140/90 mmHg; (ii) hypertensive pregnant women without proteinuria > 300 mg in 24 h and BP ≥ 140/90 mmHg; and (iii) pre-eclampsia, with proteinuria > 300 mg in 24 h and DBP ≥ 90 mmHg
Blinding	Nurses were blinded to each other's measurement readings. In addition, the software developers also did not have access to the reference blood pressure readings to ensure independent analysis.

Reporting for specific materials, systems and methods

We require information from authors about some types of materials, experimental systems and methods used in many studies. Here, indicate whether each material, system or method listed is relevant to your study. If you are not sure if a list item applies to your research, read the appropriate section before selecting a response.

Materials & experimental systems

n/a	Involved in the study
☒	☐ Antibodies
☒	☐ Eukaryotic cell lines
☒	☐ Palaeontology and archaeology
☒	☐ Animals and other organisms
☒	☐ Clinical data
☒	☐ Dual use research of concern

Methods

n/a	Involved in the study
☒	☐ ChIP-seq
☒	☐ Flow cytometry
☒	☐ MRI-based neuroimaging
